# A Multiple-Choice Task with Changes of Mind

**DOI:** 10.1371/journal.pone.0043131

**Published:** 2012-08-16

**Authors:** Larissa Albantakis, Francesca M. Branzi, Albert Costa, Gustavo Deco

**Affiliations:** 1 Department of Information and Communication Technologies, Universitat Pompeu Fabra, Barcelona, Spain; 2 Department of Psychiatry, University of Wisconsin, Madison, Wisconsin, United States of America; 3 Institució Catalana de Recerca i Estudis Avançats (ICREA), Universitat Pompeu Fabra, Barcelona, Spain; Centre national de la recherche scientifique, France

## Abstract

The role of changes of mind and multiple choices has recently received increased attention in the study of perceptual decision-making. Previously, these extensions to standard two-alternative tasks have been studied separately. Here we explored how changes of mind depend on the number of choice-alternatives. To this end, we tested 14 human subjects on a 2- and 4-alternative direction-discrimination task. Changes of mind in the participants' movement trajectories could be observed for two and for four choice alternatives. With fewer alternatives, participants responded faster and more accurately. The frequency of changes of mind, however, did not significantly differ for the different numbers of choice alternatives. Nevertheless, mind-changing improved the participants' final performance, particularly for intermediate difficulty levels, in both experimental conditions. Moreover, the mean reaction times of individual participants were negatively correlated with their overall tendency to make changes of mind. We further reproduced these findings with a multi-alternative attractor model for decision-making, while a simple race model could not account for the experimental data. Our experiment, combined with the theoretical models allowed us to shed light on: (1) the differences in choice behavior between two and four alternatives, (2) the differences between the data of our human subjects and previous monkey data, (3) individual differences between participants, and (4) the inhibitory interaction between neural representations of choice alternatives.

## Introduction

Compared to the decisions we are faced with every day, for instance what to choose for lunch in the cafeteria, or which shirt to wear, decision-making in psychophysical experiments is typically reduced to the highly simplified conditions of two-alternative forced-choice (2AFC) tasks [1], [2]. One classic example for such a 2AFC task is the random-dot motion (RDM) reaction-time paradigm, which allows investigating the temporal accumulation of evidence in order to reach a decision [3], [4], [5]. Due to the randomly moving dots, in this task the momentary amount of coherent motion is subject to stochastic fluctuations. The correct direction of coherent motion can thus be inferred more reliably the longer the motion stimulus is viewed. The behavioral data gained from these perceptual tasks, together with complementary evidence from single-cell recordings [5], [6], motivated and constrained formal models of decision-making [7], [8].

Nevertheless, by definition, 2AFC tasks neglect important features of real-life decision-making such as: (1) choices between multiple alternatives and (2) choice reevaluation after an initial decision. More recently, several authors have extended the RDM paradigm and investigated one of these two, more complex, aspects of decision-making by itself. For example, Churchland et al. [9] and Niwa and Ditterich [10] augmented the RDM task from binary to multiple choices. In particular, Churchland et al. [9] tested monkeys on a 4-alternative RDM discrimination task and compared their behavioral and neurophysiological responses to the standard binary task. In contrast, Resulaj et al. [11] considered “changes of mind” which are thought to arise from further processing of available information after an initial decision has been made. Instead of a saccadic response to the chosen target, as in the standard RDM task, human participants had to move a handle to a left or right response target. The continuous hand movement allowed for observing subjects' behavior after their initial decision had been made. Indeed, participants occasionally switched from one direction to the other, thus “changing their mind”.

**Figure 1:**
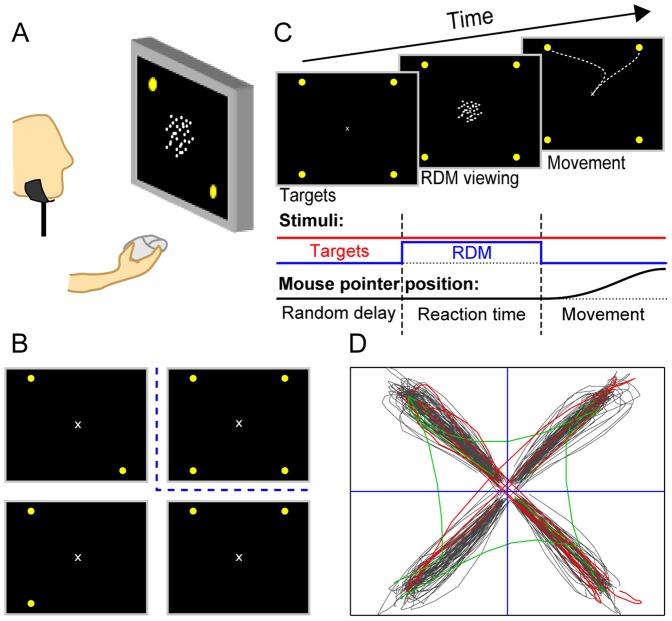
Experimental paradigm: setup, time course and conditions. (A) Participants had to decide on the net direction of coherent motion in a cloud of randomly moving dots. They indicated their choice by moving a computer mouse pointer towards the respective visual R-target on the computer screen in front of them. Yellow dots denote the R-targets. The number (two or four) and position of the visual R-targets corresponded to the possible directions of coherent motion in a particular trial. (B) Examples of possible R-target arrangements in the 2- and 4-choice experiment. In the 4-choice condition the four R-targets were placed equidistantly, each in one corner of the screen (up-right panel). For two alternatives the possible direction of motion could either be located in opposite corners of the screen (one example shown up-left), or at the same side of the screen (two examples, bottom panels). (C) To start a trial, the subjects had to click on the starting position (screen center) whereon the visual R-targets showed up on the screen, indicating the possible directions of motion. After a random delay the RDM stimulus appeared in the screen center. The motion stimulus was switched off once the mouse pointer left the starting position and the trajectory to the R-target was recorded. (D) Example traces from one participant (4-choice condition). In the majority of trials the subjects moved directly to one of the visual R-targets (black traces). Some trajectories, however, revealed a change of mind during the movement: they started towards one, but terminated at another R-target. Changes could be observed between adjacent (green) and opposite (red) R-targets.

Here, we combined these two paradigms in order to explore the extent to which changes of mind depend on the number of choice-alternatives (Fig. 1). When presented with four possible alternatives, our naive human participants showed longer reaction times and committed more initial errors (i.e. choosing the wrong response) than in 2-choice trials. The higher number of possible motion directions thus obviously led to more confusion between choices. Therefore, one might also have anticipated more changes of mind for four than for two choice trials. Yet, this was not the case, and changes of mind were similar regardless of the different numbers of alternatives. Participants neither corrected their initial decisions more often for four alternatives, although the number of initially wrong choices was much higher, nor did they make more erroneous changes of mind, although the number of possible distracters was higher in the 4-choice trials.

These findings are inconsistent with a simple race model of decision-making [12], [13] in which the evidence for each choice-alternative is accumulated independently for each choice without mutual inhibition.

In contrast, our experimental results could be fit by an attractor model of decision-making. This model was constructed by merging two biophysically-realistic attractor models for decision-making, which explained choice behavior, neural activity and changes of mind of the two preceding experimental studies [14], [15]. Characteristically, the long-term behavior of attractor models is determined by their “fixed points”, or “steady states”, which here act as decision-attractors. The decision process thus corresponds to diffusion in a nonlinear landscape of stable fixed points. In the biophysically-plausible implementation of the attractor network used here (based on [16], [17]), transitions between attractor states were gradual because of the slow kinetics of NMDA receptors. The network's behavior is thus not just dominated by its steady states, but also exhibits prolonged responses to momentary sensory inputs, during which the model effectively integrates the incoming inputs [8].

What is more, the 2- and 4-choice attractor model for changes of mind implicitly accounts for between-subject differences by an alteration of the decision threshold. A lower threshold in the model caused faster reaction times and more changes of mind. This corresponds to the strong negative correlation we found between the mean reaction times of individual participants and the tendency to change their mind.

In sum, our combined experimental and simulation approach revealed that inhibition between the different choice alternatives was necessary to account for participants' changes of mind. This study thus exemplifies how extending classic 2AFC paradigms can complement and enhance our current knowledge on perceptual decision-making.

## Methods

### Human Subjects

This study was conducted according to the principles outlined in the Declaration of Helsinki on human experimentation (WMA Declaration of Helsinki - Ethical Principles for Medical Research Involving Human Subjects, 2008) and approved by the local *Ethics Committee for Clinic Investigation (CEICs)* at the Parc de Salut de el Mar in Barcelona. Fifteen healthy young adults (10 female; mean age 22, range 19–27), right-handed and with normal vision participated in this study. One participant was excluded from further analysis because of his very poor performance (<65% overall accuracy). None of the participants had any previous experience with visual psychophysics. Each participant underwent four experimental sessions over the course of one day. Written informed consent was obtained from all participants. They received a monetary reward (30 Euros) for participating in the experiment.

### Experimental setup

Participants sat in a dark room in front of a 21-inch flat-screen cathode ray tube video monitor (Sony Trinitron Multiscan CPD-G520 21). Viewing distance from the computer screen was 40 cm (Fig. 1A). Participants placed their head on a chin-and-forehead-rest, which was calibrated individually before each experimental session. The visual stimuli were generated and data were collected using MATLAB (Mathworks, Natick, MA) and the Psychophysics toolbox-3 [18], [19] on an ASUS P5K SE/EPU computer running Microsoft Windows XP at a frame rate of 75 Hz.

### Experimental task and visual stimuli

The random-dot motion discrimination task is illustrated in Figure 1. In this task, participants were presented with a random-dot motion (RDM) stimulus that varied in the percentage of direction-coherent moving dots. Participants were asked to decide as fast and accurate as possible on the net direction of motion of the stimuli. They had to indicate their choice by moving a mouse pointer to the related response target (“R-target”, aligned with the identified motion direction). In each trial, there were either two or four possible directions in which the dots could move coherently.

Each trial began with the presentation of a gray circle of 2° diameter at the center of the screen with a small fixation-cross in the middle (the so-called “start-target”). To begin the trial, participants had to click on the start target with the mouse, and the potential response options (R-targets) appeared on the screen. The R-targets indicated the possible directions of motion. After a random delay (sampled from a truncated exponential distribution, range 0.7–1.0 s; mean 0.82 s), the fixation-cross disappeared and was replaced by the random-dot display. Upon the presentation of the RDM stimulus, participants were free to respond by moving the mouse towards the preponderant direction of motion, and clicking into the corresponding R-target. Participants had to keep the mouse pointer within a 2° diameter around the fixation-cross, until they decided to start a response. The RDM stimulus was extinguished as soon as participants moved the mouse device out of this area. The time limit to leave the starting position was 2 s (timeout #1). Once they moved out from the starting position, participants had 1 s (timeout #2) to click on one of the R-targets (within a square area of 3° edge length around the R-target). Visual feedback about the responses' accuracy (*“error”*, *“good”*) was given after each trial. They also received a “*time out*” alert message whenever they exceeded timeout #1 or #2 or “*wait for cue!*” if the mouse pointer was moved out of the start-target before the RDM stimulus was presented. Finally, *“no target hit!”* was displayed every time participants were not accurate in selecting the start target or an R-target area properly. These trials were excluded. Only trials in which one of the presented R-targets was hit within the timeout boundaries were counted as “valid trials”.

The stimuli were constructed in the following way. The RDM stimulus consisted of a multi-component pattern of small white moving dots (small filled squares with an edge length of 2 pixels). The dots appeared within a circular aperture (diameter = 5.0°) at the center of the screen. The percept of apparent dot-motion was created as follows: The stimulus consisted of three independent streams of dots that were presented alternately every three frames. With the next presentation of a particular set of dots three frames later, these dots were displaced as follows: Dependent on the respective amount of coherent motion, a certain percentage of dots were shifted in a particular direction, while the other dots were replaced at a random location. In that way, the dot positions of frame 3 for example were correlated with those of frame 6, but not with frame one, two, four, or five [4], [5], [20]. The coherently moving dots had a speed of 6.0°/s. Dot density was 16.7 dots/(deg^2^·s). We used a set of eight different coherence levels (0%, 3.2%, 6.4%, 12.8%, 25.6%, 51.2%, 76.8% and 100%).

The R-targets were constructed as yellow circles (diameter = 2.0°) located at the corners of a virtual square around the central fixation-mark (edge length 28°, and thus 19.8° distance to the center). The location of the R-targets indicated the possible directions of coherent motion in each trial. They could appear either:

- in each of the four corners of the virtual square (4-choice trials), or
- in just two of the four corners (2-choice trials).

Figure 1B illustrates the R-target locations in the 4-choice trials (top right) and three of the six possible R-target combinations for 2-choice trials. The R-targets remained present on the screen until the end of the trial.

### Experimental Sessions

Our participants underwent four experimental sessions of 30 minutes each, all in the same day, separated by a time interval of two hours. In the first three sessions, we tested the participants on the combined 2- and 4-alternative task, explained above. Each of these sessions consisted of a total of 348 trials: 232 trials with two choice alternatives and 116 4-choice trials, presented in random order. In half of the 2-choice trials the R-targets were located at opposite screen corners such as shown in the upper left panel of Figure 1B. In the other half of the 2-choice trials the R-targets were located at the same side (up, down, left, or right of the screen). The eight coherence levels were presented 32 (2-alternative) or 16 (4-alternative) times each, except for 0% which was only presented eight or four times, respectively. The 0% coherence level was presented less often to avoid frustrating participants with unsolvable trials. For 0% coherence the “correct” target was defined randomly. After the first half of trials participants could have a small break. In the beginning of each session we also included 87 practice trials to familiarize the participants with the task and to assure a satisfactory level of performance.

In the fourth experimental session, we replicated the visual arrangement used by Resulaj et al. [11] as a control to compare our simpler setup with the original binary changes of mind paradigm (“2-Top control”). Here, participants had to decide whether the coherent motion was horizontally right or left. Hence, in this fourth session participants were always presented with the same two possible motion directions and the following R-target configuration. The start-target was located at the bottom, 14° from the center, and the two R-targets at the top of the screen, 19.8° from the center, on the edges of a virtual triangle (Fig. S1A). Accordingly, in this configuration, the R-targets were not perfectly aligned to the possible motion directions. In the 2-top control we used only the first six coherence levels, and thus a total of 440 trials preceded by 88 practice trials, timeout #1 was 2.0 s, timeout #2 0.7 s. Except for that, the 2-top control was conducted in the same way as the 2- and 4-choice task described above.

Task instructions were always first given verbally and afterwards repeated visually on the video-screen. The subjects were not explicitly informed that they could change their decision during the motion response.

### Data analysis

In the analysis of our experimental data, three dependent variables were of special interest. First, we measured reaction times (RTs), corresponding to the time taken by the participants to initiate their response. More precisely, it denotes the time between the onset of the RDM stimulus and the moment when the mouse pointer left the start-target area. Second, we measured the accuracy of the responses, i.e. the percentage of correct trials. The percentage of trials in which the correct R-target was selected determined the “final performance” of the participant. Third, and more importantly for our purposes, we measured the mouse pointer trajectory at 75 Hz. Based on the mouse pointer trajectories, we determined whether subjects changed their initial decision. If a trajectory was initially directed towards one R-target, but then crossed the horizontal or vertical axes and ended at another target, this trial was counted as a change of mind. We further distinguish between “correcting changes”, which were responses that started towards an incorrect R-target, but then turned to the correct R-target, and “erroneous changes”, which turned from the correct to a wrong choice. Throughout the study the 0% coherence level was analyzed separately, as responses are neither correct nor wrong without coherent motion and the number of 0% trials was smaller than for the other coherence levels. The initial direction of the trajectory was interpreted as the participants' “initial performance”. Trajectories of trials without changes were typically straight to the target (see Fig. 1D). The initial direction and changes of mind could thus be determined reliably by their deviation from the diagonal between the center and the chosen R-target. In particular, the criteria for a change of mind were: (1) the area between the mouse trajectory and either the horizontal, or vertical axis had to exceed 2100 pixels^2^ in a screen quadrant other than the one finally selected. (2) The trajectory excursion had to exceed the start-target area by 20 pixels in a quadrant other than the one finally chosen. This quadrant then denoted the initial choice. We further rechecked the accuracy of the selection algorithm by visual inspection.

In the analysis of our experimental results, we collapsed all 2-choice trials across the different target locations for a fair comparison to the 4-choice trials, where confusion could occur between adjacent and opposite R-targets.

### Attractor model

Recently we showed that biologically-inspired attractor models can account for primate decision-making behavior and neural activity in a multiple-choice RDM task [14]. Furthermore, they also match human choice-behavior and changes of mind in a binary RDM paradigm [15]. Here we assess the conformity of a modified version of the multiple-choice model presented in [14] with the present set of psychophysical results. The network characteristics and kinetics are summarized in Table S1 (see also the original study of Brunel and Wang [16]). All default simulation parameters are listed in Table S2.

#### Network structure and connectivity

In short, the network consists of 500 leaky integrate-and-fire neurons with conductance-based synaptic responses (400 excitatory pyramidal cells and 100 inhibitory interneurons). Excitatory synaptic currents are mediated by fast AMPA and slow NMDA glutamate receptors, inhibitory post-synaptic currents by GABA_A_ receptors. External inputs are assumed to arrive only via fast AMPA receptors.

**Figure 2:**
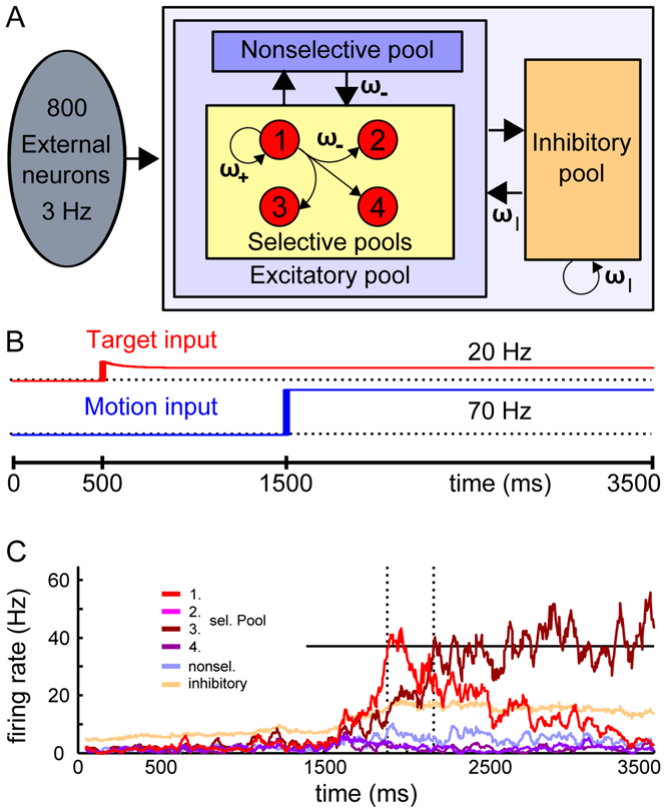
Computational model: populations, connectivity and input. (A) Diagram of the attractor model for decision-making between up to four choice alternatives. The network consists of a population of excitatory pyramidal neurons, structured into four selective pools (red, each contains 20% of the excitatory neurons) and a nonselective population, that inhibit each other through shared feedback from an inhibitory pool of interneurons (orange). Unlabeled arrows denote a connectivity of 1 (baseline). Recurrent connectivity within a selective pool is high, ω_+_ = 1.48, whereas the connection weight between the selective pools is below average ω_−_ = 0.88. Inhibitory connections have a weight ω_I_ = 1.125. The network consists of 500 neurons. (B) Time course of target and motion input to the selective populations in order to model the experimental design of the RDM task (see methods). (C) Example trial with “change of mind” for four alternatives at 3.2% coherence. The initially winning population (first threshold crossing) is overtaken by the other transient. The horizontal black line at 37 Hz indicates the threshold. Dotted vertical lines mark times of threshold crossings.

The network structure is sketched in Figure 2A. Excitatory neurons are subdivided into four selective populations or “pools” (each 80 neurons), encoding the four possible motion directions, and a fifth pool of nonselective neurons. The latter emulates the activity of surrounding neurons that are not selective to any of the four R-target directions. All excitatory neurons are connected to one pool of inhibitory neurons, which regulates the overall activity by implementing competition in the network.

The recurrent connections between neurons within one selective pool are stronger (ω_+_ = 1.48) than between the different selective pools and from the nonselective to selective pools (ω_−_ = 0.88).

As in previous studies, we used a mean-field reduction of the full spiking-neuron model to initially locate the working point of the network with respect to the two crucial bifurcations that contain the range of categorical decision-making [14], [15], [16].

#### Simulation of sensory inputs

Three types of external inputs were applied to the neural network as noisy uncorrelated Poisson spike trains. First, all neurons received a background input of ν_ext_ = 2.4 kHz, equivalent to 800 excitatory connections from external neurons firing at 3 Hz. Furthermore, two “sensory” inputs mimicking the task-relevant visual stimuli, namely the R-targets (ν_target_) and the random-dot motion stimulus (ν_motion_), were applied to the selective populations only (Fig. 2B).

Corresponding to the experimental paradigm, the target input ν_target_ was applied to two of the selective pools in the 2-choice condition and to all four selective pools to model the 4-choice condition. Departing from [14], the target input we used here was mostly flat, with only modest initial adaptation:
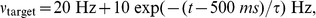
(1)where 500 ms is the onset time in the simulation and τ = 100 ms the adaptation decay time constant.

Other than the target input, the RDM input ν_motion_ was always applied to all four selective populations for the 2- and 4-choice condition. Coherent motion was simulated as a positive bias to one selective pool, balanced by a reduction of the motion input in the other three selective pools:
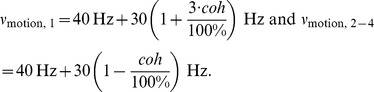
(2)Thereby, the total motion input to the network was kept constant. A motion coherence of 100% thus corresponds to a bias of 90 Hz to one selective pool, resulting in ν_motion,1_ = 160 Hz to the first selective pool and ν_motion,2–4_ = 40 Hz to the other selective pools.

#### Model fit and simulations

The output firing rates of the simulated neural populations were calculated as the average spikes per second in a 50 ms time window (shifted by 5 ms steps), divided by the number of neurons in the population. A (first) decision was reached in the simulations, when the activity of one selective pool crossed a fixed decision threshold and surpassed the other pools by at least 5 Hz. The same conditions applied for a change of mind. The threshold was assumed to be independent of motion coherence and the number of choice alternatives [5], [9]. More explicitly, one single threshold value determined first decisions and changes of mind for all coherence levels in the 2- and 4-choice conditions. To account for the mean behavioral data averaged across all 14 participants, we used a threshold value of 37 Hz. By setting the decision threshold respectively to 22 Hz or 52 Hz on the same simulated trials, we further fitted the average behavioral data of the three participants with most and fewest changes of mind according to their total number of changes. All threshold values were selected by hand with a resolution of 1 Hz, based on the overall best fit to experimental reaction times, performance, and changes of mind.

Reaction times were calculated as the time of threshold crossing plus a non-decision time t_ND_ = 330 ms [10], which also set the time limit for changes of mind (see [11] and [15] for a detailed discussion of this timeout). A t_ND_ of 330 ms is in accordance with an assumed afferent signal latency of about 180 ms for the motion signal [5], [9], plus 150 ms to account for movement initiation and execution [21], [22].

5,000 trials of 3,500 ms were run for each parameter set and motion coherence. The coupled differential equations that describe the dynamics of all cells and synapses (Table S1) were integrated numerically using a second-order Runge-Kutta routine with a time-step of 0.02 ms. The model simulations were implemented in a custom-made C++ program. Custom-made MATLAB programs were used for later analysis.

### Race model

**Figure 3:**
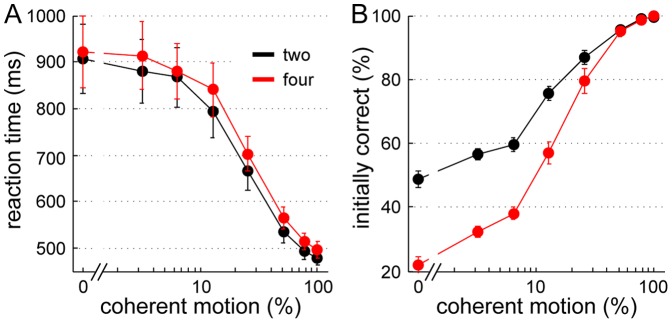
Mean reaction times and initial performance. (A) Reaction times decreased with increasing coherent motion and were larger for four alternatives. (B) Initial performance, that is the percentage of initially correct choices, started at chance level (50% for two and 25% for four choice alternatives) and increased to almost perfect accuracy for 100% coherent motion.

Apart from the biophysically-realistic attractor model, we also tested a simple race model [12], [13] on our experimental data. The race model consists of one independent accumulator for each choice-alternative, i.e. two accumulators to simulate the 2-choice condition and four accumulators for the 4-choice condition. The “first” accumulator, representing the correct direction of motion, thereby receives normally distributed random increments with mean accumulation rate k·coh and a standard deviation of σ = 70 Hz·s^−1/2^. The mean accumulation rate of the other (one or three) accumulators is zero; their increments are purely random with σ = 70 Hz·s^−1/2^. A first decision is made if one of the accumulators reaches the decision threshold and surpasses the other accumulators by at least 5 Hz. For convenience, we set the decision threshold to zero. Simulated reaction times and initial performance are thus determined by the following free parameters: (1) the accumulation factor k of the first accumulator, (2) the starting point Z of the accumulation process, and (3) the non-decision time t_ND_. These model parameters were obtained independently for 2- and 4-choice alternatives by fitting the race model to the experimental mean reaction times of all correct trials and the initial performance (Fig. 3). Parameter fits to the experimental data were first conducted by hand and then refined using a nonlinear curve-fitting procedure, implemented by the MATLAB function “fminsearch”, which minimized the summed squared errors between the simulated and experimental data. Fitting was repeated ten times for different initial conditions in order to alleviate the problem of local minima; the mean estimated parameter values and their standard deviation are given in Table 1 and were used for all further race model simulations.

**Table 1:** Race model parameters and their standard deviation.

Fit		k	σ	Z	t_ND_	ΔB	t_out_
		(Hz/s)	(Hz·s^−1/2^)	(Hz)	(ms)	(Hz)	(ms)
**A (** **Fig. 9E** **)**	2	563±42	70	–46±2	357±19	0	t_ND_
	4	673±11	70	–62.7±0.3	379±3	0	t_ND_
**B (** **Fig. 9F** **)**	2	563±42	70	–46±2	357±19	22±18	81±44
	4	673±11	70	–62.7±0.3	379±3	26±21	72±43

**Figure 4:**
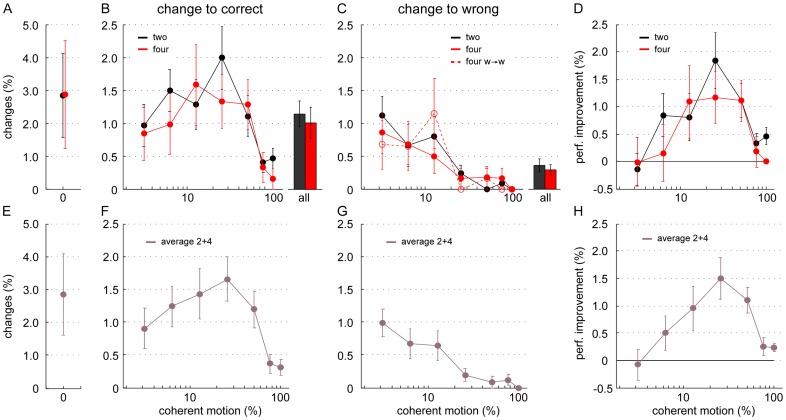
Comparison between changes of mind and performance improvement for two and four alternatives. Changes of mind are displayed as percentage of all valid trials. In the top row (A–D) the data for two and four choice alternatives is shown separately in black and red. As the percentage of changes of mind did not vary significantly between the two experimental conditions, we also plotted the average data in the bottom row (E–H) to emphasize the dependence of changing on the motion coherence. (A,E) Percentage of changes of mind at 0% motion coherence. (B,F) Correcting changes, that is changes from an initially wrong to the correct R-target. (C,G) Erroneous changes from correct to wrong. Given four choice-alternatives, also changes between two wrong R-targets occurred (w→w, dashed line in C and G). For an overall comparison, the bar plots indicate the average of all correcting (B) and erroneous (C) changes of mind for two and four choice alternatives. (D, H) Performance improvement through changes of mind (absolute difference of initial and final performance considering changes of mind). Participants' accuracy consistently improved with changes of mind, especially for intermediate coherence levels. The performance gained through changes of mind was comparable for the different number of possible choices.

Changes of mind in the race model were determined by a second threshold ΔB and a timeout for changing t_out_ in line with [11]. In a first analysis on the frequency of changing (Table 1, Fit A) we used the same threshold for changing as for the initial decision (ΔB = 0 Hz) and t_out_ = t_ND_, implementing the same strong physiological constraints that were applied to the attractor model. The race model is, however, a conceptual model and thus, other than the attractor model, does not primarily aim at proposing possible physiological mechanisms that might underlie the behavioral data. Therefore, we performed a second analysis without physiological constraints (Table 1, Fit B), where we estimated ΔB and t_out_ independently for the 2- and 4-choice condition from the experimental percentages of changes of mind (Fig. 4A–C). Here, ΔB and t_ND_ were fit to the changes of mind data using the same fitting procedure as described above for k, Z, and t_ND_, the three parameters that determine RTs and performance in the race model. Generally, fitting ΔB and t_ND_ to the changes of mind data is prone to local minima. Nevertheless, as the race model is linear, variation in the parameter values does not lead to qualitatively different simulated behavior and the mean estimated parameter values correspond to average behavioral results (Fig. S2).

We simulated 10,000 trials of 3 s for each of the six coherence levels at time steps of 1 ms using a simple Euler method in custom-made MATLAB programs.

Error bars denote SEM over all correct trials for simulated reaction times. In the case of probabilities for correct choice and changes of mind the theoretically estimated SEM was calculated according to 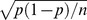 with n = 5,000 trials for the attractor model and n = 10,000 trials for the race model.

## Results

### Experimental data

To study the relation of changes of mind to the number of choice alternatives, we asked naive human subjects to perform a random-dot motion (RDM) discrimination task with two and four possible directions of motion (Fig. 1).

In addition, we intended to replicate preceding findings on changes of mind [11] with our simpler experimental setup. Therefore, we ran a separate block of trials with two alternatives in the “2-top” control condition, where the response targets (R-targets) were arranged as in [11]. The results of the 2-top control are summarized in Figure S1 and will be compared to the main 2- and 4-choice experiment in the discussion.

In all experimental conditions, we recorded the participants' choices, the time between the onset of the motion stimulus and movement initiation (reaction time or “RT”), and the movement trajectories of the mouse pointer. Changes of mind were generally defined as those traces starting towards one R-target but then changing direction and crossing the horizontal or vertical axes. Such trajectories could be observed occasionally in all experimental conditions. Notably, in the 4-choice condition subjects not only changed between adjacent R-targets, but also across the diagonal (Fig. 1D). In the following we will first report the pooled data from all 14 participants, but will show subgroups and individual results later.

#### Reaction times and choice accuracy

As shown in Figure 3, the RTs in correct trials decreased as motion coherence increased. This effect ranged from about 920 ms without coherent motion (0%) to 490 ms for the easiest coherence condition (100%) (Fig. 3A). In other words, the more dots move in the same direction the faster the response is made. More interesting perhaps is the fact that participants needed more time to decide in the 4-choice than in the 2-choice trials.

To test this statistically, we performed a within-subject ANOVA on the reaction times of all correct trials (collapsing the three sessions), excluding changes of mind, with the factors “number of choices” (two vs. four choices) and “coherence” (seven levels of coherence: 3.2%, 6.4%, 12.8%, 25.6%, 51.2%, 76.8% and 100%). The results revealed a main effect for both, “coherence” (F(6,78) = 44.13, p<.001) and “number of choices” (F(1,13) = 68.19, p<.001), but no interaction (p = .72).

Coherence also affected accuracy in a similar way: the higher the coherence the higher the percentage of initially correct responses or choices (“initial performance”) (Fig. 3B). As expected, accuracy started at chance level for 0% coherent motion and reached close to perfect performance for the highest coherence levels. At intermediate coherence levels accuracy was lower in the 4-choice than in the 2-choice condition, which reflects the lower prior probability of each choice given four alternatives.

#### Changes of mind

As explained above, changes of mind were defined as mouse trajectories that were initially directed towards one R-target, but then turned to another. They occurred in all experimental conditions, at all coherence levels and could lead to a correct or an incorrect final choice. Figure 4A separately shows the percentage of all changes of mind at 0% coherent motion. Changes there could neither be counted as correcting nor erroneous as there was no initially right or wrong choice. In Fig. 4B and 4C, correct and erroneous changes of mind are plotted for each motion coherence level as percentage of valid trials. Despite the different numbers of distracters, neither correcting changes (Fig. 4B), nor changes from the correct to the wrong choice alternative (Fig. 4C) varied significantly between the 2- and 4-choice conditions (p = .11 and p = .20, respectively). This result is particularly interesting if we consider it in the context of the different accuracy levels of these two conditions. Given four possible motion directions and low coherence levels, our participants committed about twice as many initial errors than in the 2-choice condition (Fig. 3B). They might thus have corrected their choice more often. Then again there are more competing distracters in the 4-choice condition. Yet, we did not observe more changes from the initially correct choice to a wrong R-target for four choice alternatives (Fig. 4C).

In line with Resulaj et al. [11], the percentage of changes depended on the coherence level (F(6,78) = 5.23, p<.001 for changes to correct (Fig. 4B, 4F) and F(6,78) = 6.99 p<.001 for changes to wrong (Fig. 4C, 4G)). The general shape of the curve for correcting changes (Fig. 4F) is consistent with the intuitive notion that changes to correct responses should be most frequent at intermediate difficulty. This is because for low coherences, there is only little sensory evidence that might induce a change from the initial decision. Then again, at high coherences participants already initially chose the right direction of motion. Hence, only intermediate conditions left room to find a substantial number of changes [11]. Erroneous changes overall decreased with increasing motion coherence (Fig. 4G). Importantly, changes of mind thus generally improved participants' accuracy particularly for intermediate coherences (p<.01, comparing intermediate (12.8–51.2%) against both low (3.2%, 6.4%) and high (76.8%, 100%) coherences) (Fig. 4D, 4H).

Finally, in the 4-choice condition, participants also changed between two wrong alternatives (Fig. 4C dashed line). Except for noise fluctuations the visual stimuli neither presented evidence for, nor against these changes. Note also that wrong-to-wrong changes do not affect the final performance.

In summary, there are two empirically relevant observations: First, changes of mind occurred with similar frequencies in the 2- and 4-choice condition. The probability to dismiss a correct choice decreased with higher coherence, but was apparently not influenced by more distracters. Second, changing generally improved the participants' performance for four as well as for two choice alternatives.

#### Correlations between changes of mind and mean RT for individual participants

Another interesting observation in our study refers to the individual differences in the tendency to make changes of mind. These individual differences can only be detected in experiments in which a relatively large sample of participants is tested and, perhaps, for that reason have not been addressed in detail in previous studies [4], [10], [11].

**Figure 5:**
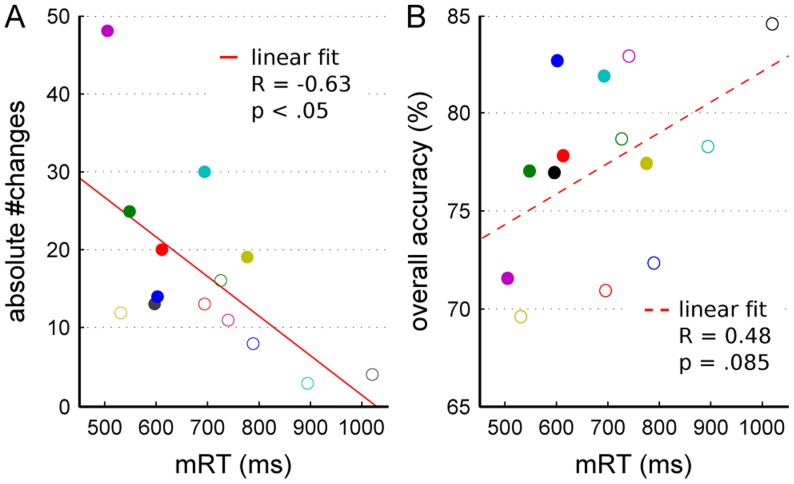
Correlation between absolute number of changes, mean reaction time and overall accuracy of individual participants. (A) The overall number of changes is plotted against the mean reaction time (mRT) of all valid trials (2- and 4-choice condition together) for each participant (colored dots and circles). (B) Relation of overall fraction of correct choices to mRT. Individual subjects are marked as in (A). On average, participants with longer reaction times changed significantly less (A) and tended to be more accurate (B). Red lines denote linear fits to the data.

Participants who made more changes tended to have faster reaction times. This can be appreciated in Figure 5A, where the overall number of changes (ONC) is negatively correlated with the mean reaction time of all valid trials (R = −0.63, p<.05, N = 14).

Furthermore, there was a trend towards a positive correlation between overall accuracy and reaction times, namely the slower the responses the higher the accuracy (R = 0.48, p = .085, N = 14). This trend is consistent with the general notion of the speed-accuracy tradeoff.

We also analyzed the correlation between participants' overall accuracy and their ONC, but found no effect there (R = −0.23, p = .418, N = 14).

Taken together, as expected, faster reaction times led to lower performance, but also increased the probability to change the initial choice. Interestingly, the relation between changes of mind and reaction times was even stronger than the well-know speed-accuracy tradeoff. We will return to the relation between changes of mind, reaction speed and accuracy in section “*Participants grouped by ONC*”. Beforehand, we will describe the computational attractor model and its fit to subjects' average choice behavior in the next part.

### Computational Models

#### Biophysically-realistic attractor model

The experimental paradigm we used here to investigate changes of mind in light of multiple alternatives is the combination of two recent experimental studies [9], [11]. Previously we showed that the results found in both of these studies can be explained by separate versions of a biophysically-realistic decision-making model with attractor dynamics [14], [15], based on the work of Brunel and Wang [16], [17]. A critical question is whether the same theoretical concepts can now also account for the 2- and 4-choice RDM results we presented above.

Generally, attractor models implement the decision process by diffusion in a nonlinear landscape of stable fixed points, which act as decision-attractors. The decision alternatives correspond to subpopulations, or “pools”, of excitatory neurons, which are selective for the respective motion directions (Fig. 2A, red). This means that they receive additional inputs if there is motion in their preferred direction. The output firing rates of these selective neural pools act as decision variables. Initially, they all fire at equal rates. With the onset of the motion input, which corresponds to the experimental RDM stimulus, the system dynamically evolves towards the decision state. In this network state, one of the selective pools fires at a high rate (winner), and the others are suppressed to low rates (losers). This “winner-take-all” competition arises through global inhibition, which is implemented by a population of inhibitory neurons connected to all neurons in the network.

The specific model we present here (Fig. 2), was developed by modifying the multiple-choice model with four selective pools presented in [14]. There, it accounted for primate decision-making behavior and neural activity in an experimental paradigm with two and four alternatives, which was very similar to our 2- and 4-choice task, but without changes of mind [9]. Notably, all parameters in the model are independent of the number of choice alternatives. Solely the target input distinguishes between conditions in the model, in the same way as the visual R-targets determined the possible motion directions in the experiment. For two choice-alternatives the target input was applied to two selective populations, while for four alternatives, all four selective pools received the target input. Corresponding to the random-dot stimulus, the motion input was always applied to all four selective populations, independent of the number of alternatives (Eq. 2). A (first) decision was noted in the attractor model, when one of the firing-rate transients crossed the decision threshold (37 Hz). If the firing rate of an initially-losing selective pool subsequently exceeded the same decision threshold and surpassed the other pools by at least 5 Hz, that trial was considered a change of mind (see Fig. 2C for an example trial).

#### Attractor model fit to average choice behavior

**Figure 6:**
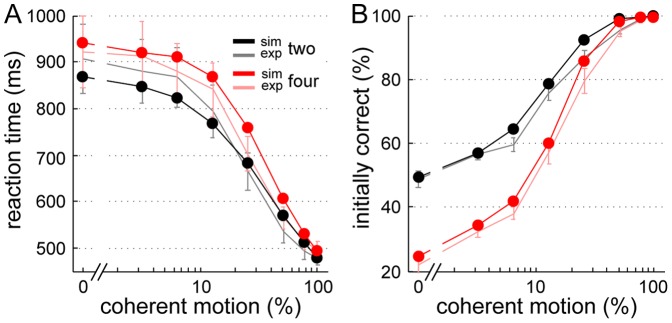
Comparison between simulated and experimental reaction times and accuracy. (A) Reaction times simulated with the attractor model include a non-decision time (t_ND_) of 330 ms. (B) Accuracy of the attractor model. The respective motion input ν_motion_ for a given level of coherence was set according to equation 2 in the methods section. The initial decision was determined by a firing rate threshold of 37 Hz. For comparison the experimental data from Figure 3 is shown again in lighter colors. The model fits experimental reaction times and performance well. The difference in RT between 2- and 4-choice trials is only slightly overestimated, so is the accuracy. Error bars of simulated data (SEM) are mostly hidden behind the dot markers.

As can be appreciated in Figure 6, the attractor model matches the experimental reaction times and accuracy well for the different coherence levels. In comparison to the primate study [9], the differences in reaction time between experimental conditions were less pronounced for our human participants and their accuracy was somewhat worse (see discussion). To adapt the model accordingly, we primarily changed three parameters: first, the connection strength between the selective pools now has no spatial component and is equal to ω_−_ for all selective pools. Second, the target input before motion onset (500 to 1,500 ms) was set much smaller, which lessened the difference between the 2- and 4-choice conditions. Third, the number of neurons in the network was reduced from 2,000 to 500. This mainly decreased the performance to a level comparable with our subjects' accuracy (Fig. 6B). Still, the model somewhat overestimated the difference in reaction time between the 2- and 4-choice trials and the simulated performance slightly exceeded the experimentally-observed accuracy. Nevertheless, these model adjustments hint at a possible cause for the differences between human and primate behavior. Roughly speaking, the model adaptations described above are congruent with less practice (see discussion).

Most importantly for our purposes, the multiple choice attractor model is able to produce changes of mind in the same way as the 2-alternative attractor model [15], which was used to fit the binary RDM experiment of [11], namely with a comparatively high motion input and a low decision threshold. Both, high inputs and a low decision threshold, generally lead to faster reaction times and less accuracy in the attractor model and thus correspond to pressure for speed in the experiment. Indeed, with a mean-field reduction of the full spiking-model, we have shown in [15] that this input-dependent, speed-accuracy tradeoff arises from a shift of the dynamical working point in the attractor landscape of the network. With higher inputs the system shifts closer to a bifurcation where a new attractor appears, which allows for two neural populations firing at elevated rates. This means that after this bifurcation, decision-making is no longer unambiguous. The system there shows multi-stability between the decision attractors (corresponding to specific decisions) and the ambiguous, symmetric attractor (corresponding to two neural populations firing at elevated rates). Interestingly, as a consequence, more changes of mind emerge the further the system is pushed towards this bifurcation. This is because in the proximity of the bifurcation, for high inputs, it becomes more likely that two selective populations reach firing rates close to the decision threshold, which facilitates changes of mind.

**Figure 7:**
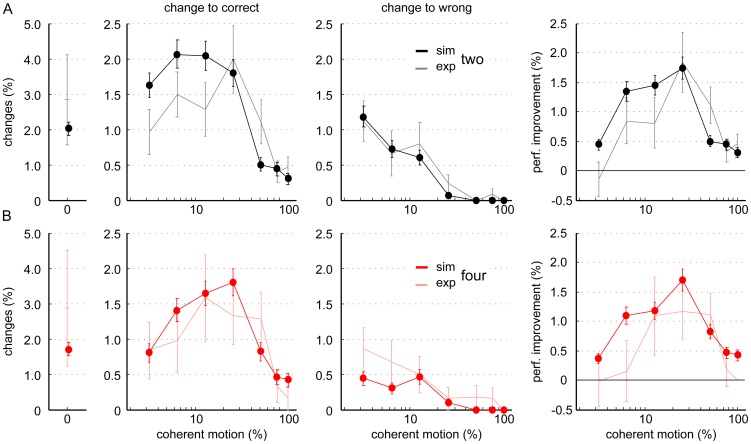
Simulated changes of mind using the attractor model. A model trial was counted as a change of mind, if, after the initial threshold crossing (first choice), the firing rate of an initially-losing selective pool surpassed the other pools and crossed the decision threshold. Changes of mind are displayed as percentage of all valid trials. The attractor model generally replicated the frequency of changes observed experimentally for two (A) and four (B) alternatives. The experimental results of Figure 4 are plotted in lighter colors for comparison. Congruent with our experimental findings, the attractor model predicted similar percentages of changes in the 2- and 4- choice condition. Only at the lowest coherence level (3.2%), even fewer changes occurred for four choices, than were observed experimentally. Besides, in the model, changing improved the performance somewhat more for low coherence levels in the same way for the 2- and 4-choice condition (right column).

Importantly, this principle applies in the same way for the 4-alternative version of the attractor model. Notably, in addition to RTs and choice accuracy, the 2-and 4-choice attractor model also fits our experimental results on changes of mind well. Even the coherence dependence of the simulated changes of mind matches that of the experimental changes of mind (Fig. 7). Moreover, in the attractor model, changing also improves the performance the most for intermediate coherence levels (Fig. 7, right column). It is only for low coherences that the absolute performance improvement is somewhat overestimated by the computational model. This is because for low coherences slightly more correcting changes were predicted by the model in the 2-choice trials, while in the 4-choice trials fewer erroneous changes occurred in the simulations than in the experiment.

Overall the attractor model predicts not more, but rather fewer changes for four choice-alternatives than for two (Fig. 7) consistent with our findings. This can theoretically be explained by the global inhibition in the network. If the target input is applied to four and not just two pools, inhibition, and thus competition, in the model increases. More competition has the opposite effect as higher inputs: it becomes less likely that two selective neural pools both reach firing rates close to the decision threshold, which is required for a change of mind.

In sum, our experimental results, including the changes of mind, are in good accordance with the attractor model of decision-making and thus explained by the same theoretical concepts as preceding studies.

#### Participant subgroups according to ONC

**Figure 8:**
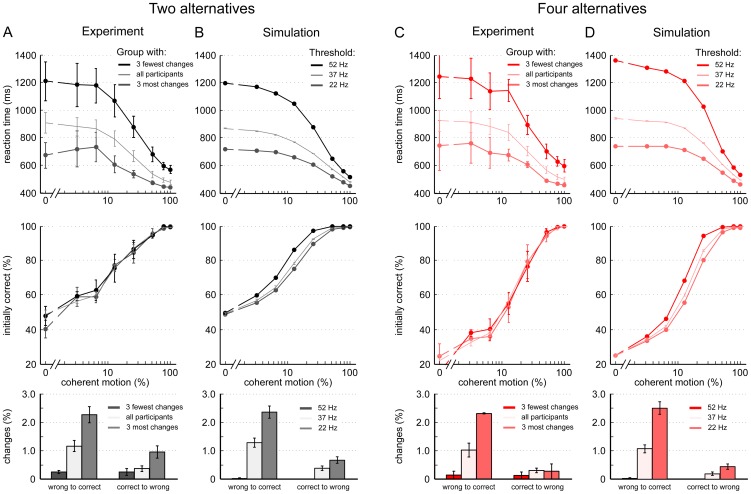
Threshold variation in the attractor model accounts for differences in choice behavior of participant subgroups. (A,C) Average experimental data of the three participants with most and fewest overall changes of mind. The experimental results of all participants (Fig. 3) are plotted in lighter colors without dots for comparison. Reaction times (top row) in both the 2- and 4-choice conditions varied substantially between the subgroups: participants with the fewest changes had much higher reaction times. However, the initial performance (middle row) was almost identical between the two subgroups. The overall percentage of correcting and erroneous changes is displayed in the bottom row. (B,D) Simulated data. Just by modifying the decision threshold, the attractor model accounted for the quantitative differences in choice behavior of the two participant subgroups. With a lower threshold (22 Hz), reaction times were faster (top row) and more changes occurred (bottom row), fitting the experimental subgroup with the most changes. The opposite is true for a higher decision threshold (52 Hz), fitting the subgroup with the fewest changes. As in the experiment, the simulated performance (B,D, middle row) varied only slightly with threshold alteration. Apart from the decision threshold, all other model parameters were kept constant. For a comparison of the changes of mind frequency for different coherence levels see Figure S3.

Another benefit of the theoretical model is that it can help to elucidate the source of variability across individual subjects. In the following, we focus on the average behavioral data of the three subjects with the most and fewest overall number of changes (ONC). As shown in the top row of Figure 8, for both, two and four choice alternatives, the reaction times between these two subgroups varied significantly (between-subject ANOVA, p<.05). The three participants with the most changes of mind responded more rapidly, those with the fewest changes of mind took longer to respond. This result corresponds to the negative correlation between ONC and mean RTs that we found across individual participants (Fig. 5). Again, similar to the analysis of individual subjects, no difference was found between the subgroups' accuracy levels (Fig. 8, middle row). The overall percentage of correcting and erroneous changes for the two subgroups is displayed in the bottom row of Fig. 8A, 8C. The data for all participants is also given for comparison.

Notably, in the computational model an adaptation of the decision threshold was enough to capture the subgroup differences described above (Fig. 8B, 8D). Decreasing the threshold from the original value of 37 Hz to 22 Hz produced faster RTs and more changes of mind in the 2- and 4-choice condition. Increasing the threshold to 52 Hz had the opposite effect. Moreover, this simple adjustment simultaneously fitted the quantitative differences of reaction times and changes of mind between subgroups remarkably well. This can further be appreciated in Figure S3, where the coherence dependence of the simulated changes of mind for 22 Hz and 52 Hz is compared to that of the two subgroups with fewest and most changes of mind. In addition, due to the nonlinear character of the attractor model, varying the decision threshold by as much as 15 Hz had only minor effects on the model's accuracy, in line with the experimental performance, which hardly differed between participant subgroups (Fig. 8, middle row).

As explained in the last section, in the nonlinear attractor network the relation between RTs, performance, and changes of mind is further dependent on the dynamical working point of the system. By contrast to linear decision-making models, it can thus in principle also be regulated by the common inputs to both selective neural populations [15], [23]. Here we focused on the decision threshold, as it is the mechanism most commonly associated with the speed-accuracy tradeoff (see discussion). Yet, adjusting the common selective inputs in addition to the decision threshold might enable a closer match of simulated and experimental initial performance. Besides the common selective inputs, also stronger recurrent connections influence the dynamical working point and would lead to faster reaction times and lower performance. Stronger recurrent connections would, however, lead to *fewer* changes of mind in the model, contrary to regulating the speed-accuracy tradeoff through the decision-threshold and common selective inputs. This is because the attractor landscape would become “steeper” and, consequently, escaping the attractor after a first decision would become more difficult.

Taken together, solely by an alteration of the decision threshold the nonlinear attractor model can largely explain the “changes-speed-accuracy” relation we observed experimentally in the behavioral variability of individual participants and subgroups.

#### Race model predictions on changes of mind

The question of how changes of mind depend on the number of choice alternatives is not at all trivial. Intuitively, but contrary to our experimental findings, one could have assumed more changes of mind for more choice alternatives because of more confusion between the higher number of possible choices.

In fact, this notion corresponds to the so-called “race model” [12], [13], a simple conceptual model of decision-making where the evidence for each choice alternative is integrated independently without interaction between the accumulators. In the following we will show that while a simple, linear implementation of the race model can be fitted to the mean reaction times and accuracy of our participants, it is not able to account for our experimental findings on changes of mind.

**Figure 9:**
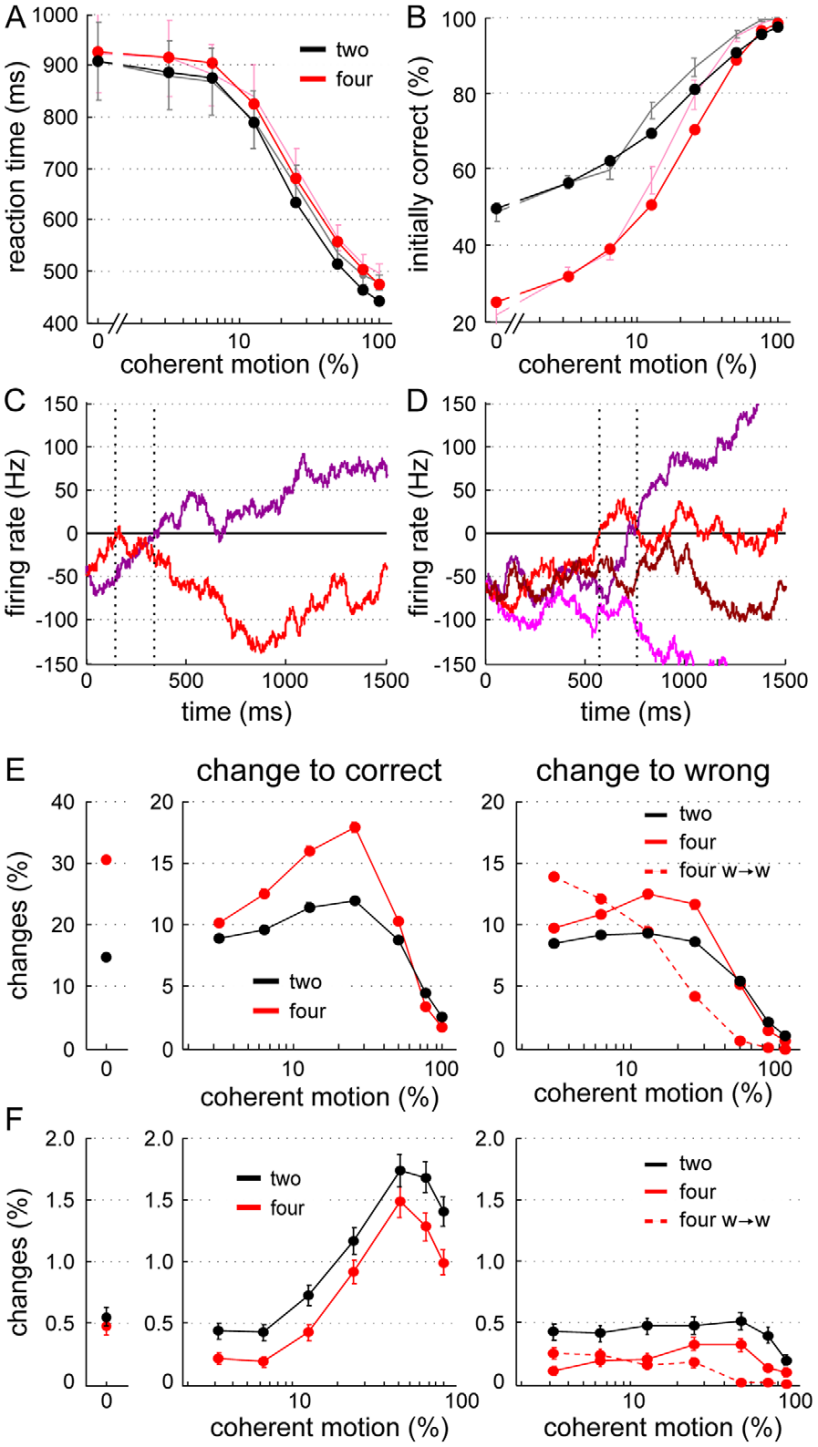
Reaction times, performance, and changes of mind simulated with a simple race model. (A) Reaction times and (B) initial performance obtained from the race model. The race parameters were estimated by fits to the experimental data and are given in Table 1. For comparison, the experimental data from Figure 3 is shown in lighter colors. The initial performance for high coherence levels is somewhat underestimated by the race model. (C, D) Example trials of a simulated change of mind at 6.4% motion coherence in the 2- and 4-choice conditions with ΔB = 0 and t_out_ = t_ND_. (E) Changes of mind from the race model for the same physiologically-plausible conditions as used in the attractor model (see Table 1, Fit A). That is, the threshold to detect changes of mind was the same as the decision threshold (ΔB = 0) and the timeout for changing corresponded to the estimated non-decision time (t_out_ = t_ND_). With these assumptions, the race model predicts ten times more changes than were observed experimentally. Moreover, in the race model, changes of mind are then more frequent for four than for two choice alternatives. (F) Changes of mind with fitted parameters for the change threshold and timeout (see Table 1, Fit B). By setting the change threshold much higher than the decision threshold and using a higher change threshold for four than for two choice alternatives, the percentage of changes of mind produced with the race model is now in the right order of magnitude. Yet, the dependence of changes on the motion coherence does not match our experimental observations (Fig. 4, see text for details). Changes of mind are displayed as percentage of all valid trials.

The model's reaction times and initial performance shown in Figure 9A and 9B were obtained by simulating a race to threshold (set to 0 Hz) of two or four independent, linear, noisy accumulators. The accumulator corresponding to the correct choice has a mean accumulation rate of k⋅coh. The other accumulators receive only noisy inputs. The accumulation factor k, the starting point Z, and the non-decision time t_ND_ are listed in Table 1. They were estimated through the experimental data as described in the Methods section and Figure S2. Except for a slight underestimation of the initial performance at high coherences, the race model captures the initial responses of our participants well.

To produce changes of mind with the race model, we introduced a second threshold ΔB and a timeout t_out_ for changing. Previously, Resulaj et al. [11] extended a binary diffusion model in the same way to account for the changes of mind in their 2-alternative RDM task. Here, we analyzed the predictions of the race model on changes of mind using two sets of parameters ΔB and t_out_ (Table 1, Fit A and B). First, we were interested in the race model's predictions under the same physiologically plausible assumptions that we applied to the attractor network: (1) the threshold for changing is the same as the decision threshold for the initial choice (ΔB = 0 Hz), and (2) the timeout for changing is determined by the non-decision time (t_out_ = t_ND_) (Table 1, Fit A). One example trial with change of mind is displayed in Figure 9C and 9D, respectively for the 2- and 4-choice condition. As shown in Figure 9E, with the above assumptions the race model produces about ten times more changes than we observed experimentally. Most importantly, however, simulated changes of mind are overall more frequent for four choice alternatives than for two, even though the starting point Z was initially lower for four than for two choice alternatives.

In a second analysis (Table 1, Fit B), we directly estimated ΔB and t_out_ from the experimental percentages of changes of mind, again independently for two and four choice alternatives. Figure 9F depicts the resulting simulated changes of mind. Although the percentage of changes is now in the right order of magnitude, their dependence on the coherence level does not match our experimental observations (compare Fig. 4A–C). Note further that the fitted ΔB is now higher, and t_out_ shorter for four alternatives than for two, to compensate for the higher confusion in the case of four accumulators.

In conclusion, the conceptual race model without interaction between accumulators can replicate the reaction times and initial performance of the first decision in our 2- and 4-choice RDM experiment. It fails, however, to account for subsequent changes of mind.

## Discussion

In this article, we presented human behavioral data from a 2- and 4-alternative random-dot motion discrimination task. Our participants reported their choice by moving a mouse pointer on the computer screen from a central start-target to one of the up-to-four response targets in the screen corners. In this setting, we were able to observe occasional changes of mind in participants' movement trajectories. These changes of mind are supposedly based on information that was still unprocessed at the time of the first decision [11]. A multi-alternative attractor model for decision-making that incorporates further processing of evidence after the initial decision reproduced the experimental changes of mind and the general choice behavior of our participants, while a simple race model failed to account for our experimental data.

In the following we will discuss our results with respect to the preceding studies that built the groundwork for our experiment. In particular, we will compare (1) the 2- and 4-choice RDM task to the binary paradigm [11], (2) differences in the behavior of individual participants, (3) human versus primate choice behavior [9], and (4) different modeling approaches.

### Comparison to binary changes of mind

Our findings on changes of mind in the 2- and 4-choice experiment are consistent with all relevant aspects of the original study on binary changes of mind [11]: Changes leading to correct responses were most frequent for intermediate coherences, erroneous changes decreased with coherent motion, and accuracy improved with mind-changing. The present study thus showed that the general principles underlying changes of mind extend to multiple-choice decision-making.

Under closer inspection, however, in comparison to the three participants tested by Resulaj et al. [11], our participants changed less often, had longer reaction times, and showed more between-subject variability. These quantitative, behavioral differences can easily be explained by two alterations in the experimental setup: first, the 2- and 4-choice task was *per se* more difficult because of the different numbers of choice alternatives. Similarly, the dot-motion here ran along the diagonals, in the direction of the R-targets and not simply to the left or right. Second, we simplified the experimental setup compared to [11]. Instead of moving the handle of an elaborate vBOT manipulandum [24], our subjects used a standard computer mouse to indicate their choice.

To distinguish the effects of the simplified setup from actual task differences, we tested our subjects on the 2-top control (Fig. S1). There, they were presented with the same target configuration as in [11] and horizontal dot-motion. This led to ∼250 ms faster RTs for low coherences and significantly more correcting changes of mind (repeated measure ANOVA, p<.05) compared to the 2-choice condition of the main experiment, which was randomly mixed with 4-choice trials (Fig. S1). Interestingly, the participants' accuracy and erroneous changes of mind in the 2-top condition were basically identical to the 2-choice condition of the main experiment.

In the 2-top control our participants still changed on average less than those of Resulaj et al. [11] and the variability between subjects was higher. Both of these effects might result from less pressure to respond fast. As moving the mouse pointer was probably easier than moving a handle in space, the same timeouts might have been less urging in our case. Nevertheless, all of our participants responded substantially faster and less accurately than subjects who performed a similar 3-alternative RDM task and were free to respond without a time limit [10].

Besides, the above comparison between the two distinct experimental paradigms (Fig. S1) further shows that, while the number of changes observed in our study is comparatively low, it is still sensitive enough to conclusively detect differences and similarities across entirely different experimental sessions.

In sum, a variable number of alternatives with coherent dot-motion along the diagonals led to longer reaction times and less correcting changes of mind. Still, the basic principles of changes of mind extend to the case of multiple alternatives.

### The “change-speed-accuracy” tradeoff

Due to the large number of participants we tested, we were able to evaluate the correlation between accuracy, reaction time, and changes of mind quantitatively (Fig. 5 and 8). Resulaj et al. [11] already noted that their participants changed less when they were asked to respond more slowly. Indeed, we found a negative correlation between the overall number of changes and the mean reaction time across participants. This correlation was significant and thus even stronger than the negative trend we found between mRTs and the mean accuracy, which corresponds to the established concept of a speed-accuracy tradeoff. A comparison between the three participants with the most and fewest overall changes of mind revealed the same trends.

Notably, the attractor model could largely explain the subgroup differences with different decision thresholds. In theoretical models of decision-making, threshold adaptation is usually associated with a within-subject speed-accuracy tradeoff [4], [25], [26]. Here we suggest that the flexibility to reevaluate an initial choice might be regulated via the same mechanism, applying to across- as well within-subject effects. In the attractor model, the threshold for the initial decision is the same as the threshold for subsequent changes of mind. Corresponding to our experimental findings, adapting the decision threshold thus provides a direct relation between changes of mind and the speed-accuracy tradeoff. This correlation is lost, however, if different, independent thresholds are used to determine initial decisions and changes of mind, as in the binary diffusion model proposed by Resulaj et al. [11], or the race model with independent thresholds of Figure 9F. In these models, decreasing only the second threshold would lead to more changes of mind, leaving RTs and performance unaltered. For this reason, and because of the race model's generally poor match to our experimental data, we did not attempt to fit it to the subgroup data.

In nonlinear attractor models the speed-accuracy tradeoff can further be adjusted by the common synaptic input to the selective populations [15], [23]. As higher inputs shift the working point of the system closer to a bifurcation where two neural populations can fire at elevated rates, higher inputs also produce more changes of mind [15]. Higher common inputs thus provide a second implicit link between changes of mind and reaction times, corresponding to our experimentally observed changes-speed-accuracy relation.

As these regulatory mechanisms are implicit in the attractor network, they further imply the existence of a within-subject changes-speed-accuracy relation, analogous to our findings across subjects.

Furthermore, the suggested connection of changes of mind and the speed-accuracy tradeoff through a common decision threshold and/or higher common inputs moreover has a crucial implication on the nature of changes of mind: While there might generally be a strategic advantage associated with changes of mind, our modeling effort in the present study and [15] demonstrates that changes of mind are a phenomenon that can arise naturally in a neural decision-making network given a pressure to respond fast. Speed pressure, corresponding to a low decision threshold and/or common selective inputs will automatically result in changes of mind as observed experimentally, without the need to postulate a strategic advantage.

Taken together, the across-subject analyses of our experimental data imply a regulatory connection between reaction times and changes of mind, with weaker effects on performance. Contrary to conceptual linear models, the nonlinear attractor model offers an implicit explanation of this relation through the decision threshold and common selective inputs.

### Comparison of human choice behavior to previous findings with primate subjects

In their groundbreaking study on multiple-choice decision-making, Churchland et al. [9] measured the neural activity of macaque monkeys performing a multi-alternative RDM task. Here, we adopted their combined 2-and 4-choice paradigm, except that our participants indicated their choice by a continuous movement instead of a saccade, in order to observe changes of mind.

Interestingly, the difference in reaction time between the different numbers of alternatives was much more pronounced for primates than for our human subjects, and the primates generally performed the task more accurately [9]. Decision-making in the RDM task should nevertheless be based on the same principles in both species, with the difference that monkeys are usually trained for months on a psychophysical task, before the final experiment is conducted.

Notably, the attractor model suggests that training effects can indeed largely explain the distinct behavior between species. This can be concluded from differences in crucial network parameters between the model implementation we used here and a previous version that was applied to the monkey data [14]. The previous implementation of a multi-alternative attractor model [14] could account for all relevant aspects of Churchland et al.'s [9] findings, including the differences in neural activity between two and four choice alternatives. To this end, graded spatial connectivity had to be assumed between the selective neural populations that encode the different motion directions. We suggested in [14] that such a spatial connectivity component might have emerged during training through Hebbian learning.

In contrast, when fitting the behavior of the untrained human participants, we did not find any advantage of such a spatial-connectivity component. Therefore, we dropped it here and used equal weights between all selective neural populations. Moreover, to approximate the somewhat worse performance of our human subjects, we reduced the network size from 2,000 to 500 neurons. This increased finite size noise in the model and thereby reduced its accuracy. As the attractor model for simplicity assumes full connectivity between neurons, the number of network neurons cannot be taken literally. Nevertheless, it is a plausible prediction of the model that with training substantially more neurons might be recruited to encode possible motion directions. Comparing fluctuations in neural activity (e.g. the fano factor) before and after extensive training on the RDM task might provide an indirect measure of the relative number of neurons involved in the task through the amount of finite size noise present in the network.

Overall, the comparison of the computational models revealed that human and primate decision-making can be accounted for by the same theoretical mechanisms. Accordingly, our experimental results generally agree with Churchland et al.'s [9] findings for primates, despite deviating behavioral observations. Moreover, the “monkey-model” [14] would predict even fewer changes for four than for two choices. It would be interesting to see if the overall frequency of changes of mind in trained monkeys indeed varies more between the different experimental conditions. Single cell recordings of the neural activity during changes of mind would further allow testing the attractor model's prediction on neural activity during changes of mind (Fig. 2C) and the prediction of a common threshold for changes of mind and the first decision. Training primates to perform the manual RDM task could thus lead to great insights into the nature of changes of mind and decision-making in general.

### Intuition and possible models for changes of mind

In our experimental setup the different *a priori* probabilities to choose a particular direction make it difficult to form an intuition about the dependency of correcting and erroneous changes of mind on the number of alternatives. Strikingly, the probability to discard an initially-correct R-target seems to depend only on the given level of evidence, which here means the coherence level in the motion stimulus: Erroneous changes of mind occurred independent of the number of alternatives and target locations relative to the motion direction (Fig. 4C and Fig. S1F). Furthermore, participants corrected their initial errors in similar percentages of trials for two and four choice alternatives, although the number of initial errors was much higher in the 4-choice condition (Fig. 3B and 4B). Here it is interesting to note that more choice corrections occurred for the simpler binary paradigm with only left/right choices (Fig. S1E). Even if the prior probabilities are taken into account, there is no simple intuitive explanation for these findings.

Notably, the attractor model of decision-making produced the changing behavior of the 2- and 4-choice experiment through its global inhibition, while the threshold and all other network parameters were identical across conditions.

Attractor models are, however, not the only decision-making models that are generally capable of producing changes of mind. Resulaj et al. [11] extended a linear diffusion model by an independent, second threshold that determined changes of mind. This model could then be fitted to their experimental findings on changes between two alternatives (left/right). In Figure S4A–C we show that such a diffusion model is generally capable of fitting our experimental data from the 2-choice condition. Because in the diffusion model it is the difference in evidence between two choices that is integrated, an extension to more than two alternatives is not straightforward.

Here the race model without interaction between accumulators has the advantage that it can easily be extended to any number of choice alternatives [27]. Churchland et al. [9], additionally, accounted for their 2- and 4-choice data by a race between two independent diffusion models. Comparing a simple race model to our experimental data we found, however, that an independent race between accumulators is inconsistent with our findings on changes of mind. Without inhibition between the different choice alternatives, more possible choices lead to more changes of mind (Fig. 9E). Even when compensating for this property of the race model through number-of-choice dependent thresholds and timeouts for changing, the resulting coherence dependence of the changes of mind did not match our experimental observations (Fig. 9F). Consequently, we claim that our experimental findings have ruled out the race model as a conceptual description of the neural decision process during the RDM task.

As a first approximation to mutual inhibition between four accumulators, we further tested two adapted versions of the 4-choice race-model assuming positive and negative accumulation rates, but independent noise for all accumulators (Fig. S4D–F). A race of one accumulator with positive accumulation rate +k for one pool and negative rates −k/3 for the other three accumulators already yields substantially better fits to our 4-choice experimental data on changes of mind than the classic race model. Nevertheless, a “true” extension of the diffusion model to multiple alternatives would require a connectionist implementation with explicit mutual inhibition, i.e. anticorrelated noise, between the neural accumulators.

Niwa and Ditterich [10] have recently proposed an extension of the diffusion model to three alternatives through weighted feed-forward inhibition. It is probable, that a similar 4-alternative version of this connectionist diffusion model could reproduce our experimental observations, especially if the threshold for changes of mind were a free parameter as in [11].

Here it has to be emphasized again, that in the physiologically inspired attractor model the change threshold is the same as the decision threshold, which implicitly gives rise to the experimentally observed “changes-speed-accuracy” relation. Moreover, all network parameters in the biophysically realistic attractor model are independent of the number of possible alternatives, in contrast to the conceptual models where two parameter sets are fitted independently to the 2- and 4-choice case. Notably, in the attractor model, the sensory input that represents the number of R-targets present in the 2- and 4-choice case is sufficient to explain subsequent behavioral differences between conditions.

It would be very interesting to see how our experimental results on changes of mind between multiple alternatives would further constrain connectionist diffusion models [10] and other existing multiple-choice decision models [28], [29].

Taken together, while more alternatives lead to more initial errors, we have discovered that changes of mind improve the initial performance by the same percentages for two and four choice alternatives and mainly for intermediate coherences. Through the combined approach of experiment and theoretical models, we were able to show that inhibition between the different choice alternatives is necessary to account for our experimental findings. Moreover, we were able to further establish and explain a relation between changes of mind, reaction speed, and accuracy over the decision threshold. Finally, we proved that studying changes of mind in multiple-choice decision-making paradigms can indeed extend our understanding of the neural computations underlying decision-making and could further help to distinguish between theoretical models in the future.

## Supporting Information

Figure S1 **Choice behavior in the 2-top control condition.**
(PDF)

Figure S2 **Variation in the race model**'**s estimated parameter values.**
(PDF)

Figure S3 **Attractor model with adapted thresholds compared to frequency distributions of changes for the three participants with most and fewest changes.**
(PDF)

Figure S4 **Diffusion model fit to behavioral data of the 2-choice condition and 4-choice approximations.**
(PDF)

Table S1 **Attractor model summary (according to guidelines in Nordlie et al. (2009)).**
(PDF)

Table S2 **Default parameter set used in the integrate-and-fire simulation (attractor model).**
(PDF)
