# Supplementary material for: A Multiple-Choice Task with Changes of Mind
Source: PLoS One. 2012 Aug 16;7(8):e43131. doi: 10.1371/journal.pone.0043131 (PMC3420910; doi:10.1371/journal.pone.0043131)
Supplement: Figure S1 — Choice behavior in the 2-top control condition. (PDF) [file pone.0043131.s001.pdf]

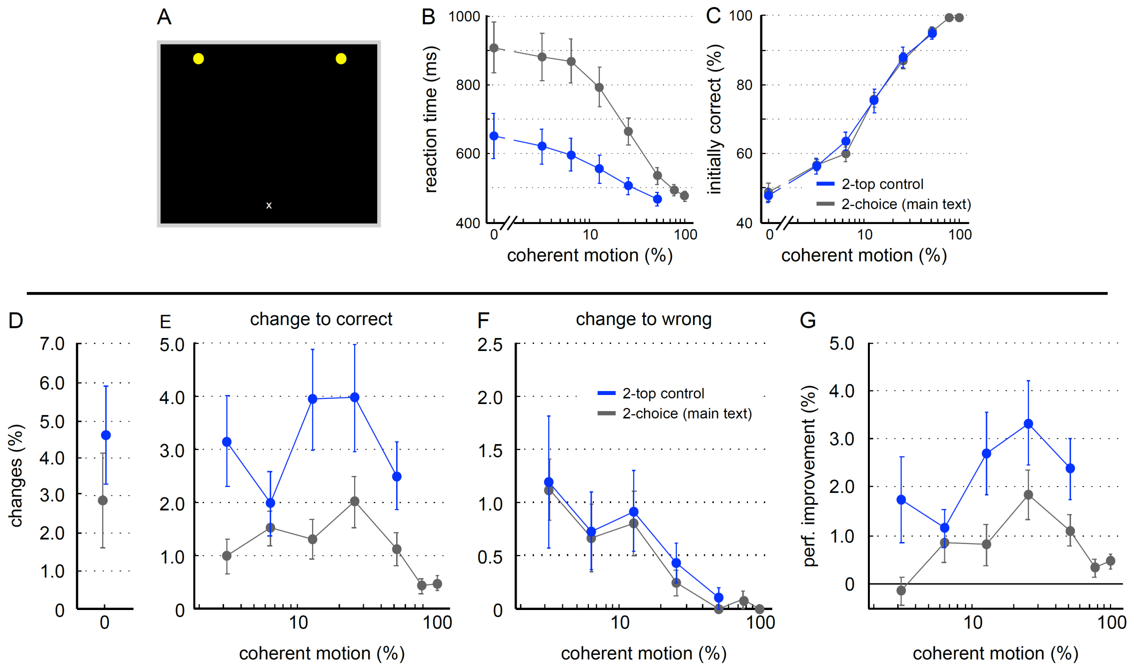

Figure S1: **Choice behavior in the 2-top control condition.** (A) In a separate block of trials we replicated the experimental design of Resulaj et al. (2009) with two R-targets at the top of the screen and the starting point at the bottom (“2-top” condition) with our simpler setup using a computer mouse instead of a handle. (B) Reaction times and (C) performance averaged across all 14 participants. The 2-alternative condition from the main text is plotted in gray for comparison. While the reaction times in the 2-top condition were much faster, than for two alternatives in the 2- and 4-choice paradigm, performance is basically identical. Note that in the 2-top condition, the possible motion directions were always horizontal (left or right), while in the main experiment coherent dots moved along the diagonals. (D-G) As Fig. 4 main text. (D-F) Changes of mind as percentage of all valid trials. Changes at 0% motion coherence (D) and correcting changes (E) were more frequent in the 2-top condition than for two alternatives in the main experiment. Erroneous changes (F), however, happened with the same frequency. Thus, the performance improvement (H) was greater in the 2-top condition. The performance improvement is the absolute difference of the initial and final performance, i.e. considering changes of mind. Error bars denote SEM.
