# Supplementary material for: A Multiple-Choice Task with Changes of Mind
Source: PLoS One. 2012 Aug 16;7(8):e43131. doi: 10.1371/journal.pone.0043131 (PMC3420910; doi:10.1371/journal.pone.0043131)
Supplement: Figure S2 — Variation in the race model's estimated parameter values. (PDF) [file pone.0043131.s002.pdf]

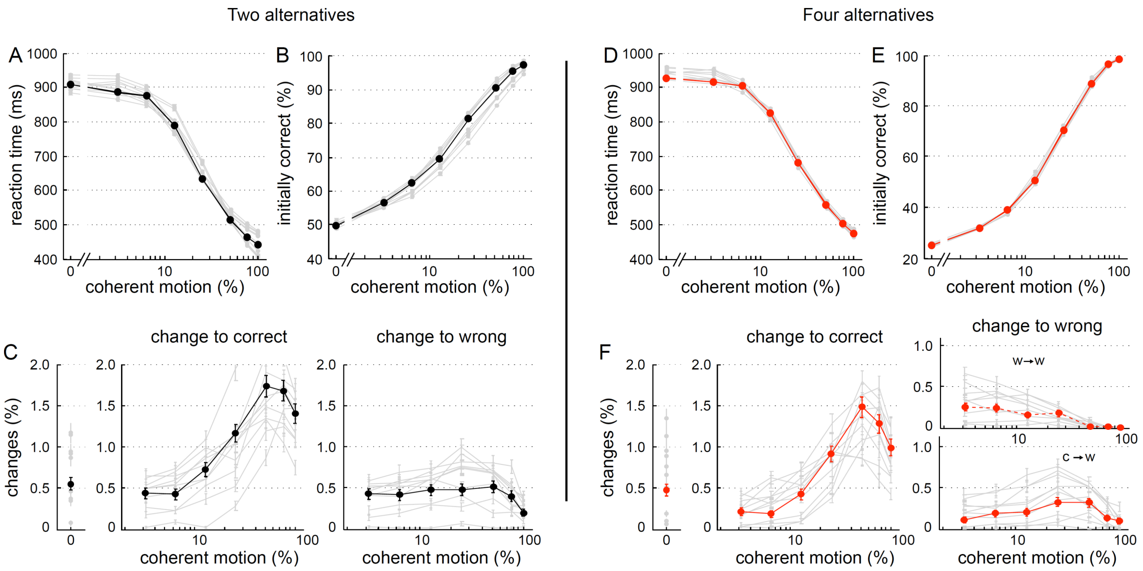

Figure S2: **Variation in the race model's estimated parameter values.** (A, D) Shown in gray are the simulated RTs and (B, E) initial performance of the race model for the ten parameter sets (k, Z, and t_{ND}) with different initial conditions that were obtained from fits to the experimental RTs and initial performance. For comparison, the simulated behavioral data of the mean estimated parameter values (Table 1, Fit B and Fig. 9) are displayed in black and red, respectively, for two and four choice alternatives. The variation in RTs and initial performance for the different parameter sets is relatively small. (C, E) Simulated changes of mind for the ten parameter sets (ΔB and t_{out}) estimated independently for the 2- and 4-choice condition from the experimental percentages of changes of mind. The upper right panel in (F) shows changes from wrong to wrong (w → w), the lower right panel displays erroneous changes from the initially correct to a wrong choice. Fitting ΔB and t_{ND} to the changes of mind data is prone to local minima. Nevertheless, as the race model is linear, variation in the parameter values does not lead to qualitatively different simulated behavior and the mean estimated parameter values (black and red) correspond to average behavioral results. We simulated 10,000 trials of 3 s for each coherence level. Error bars denote SEM.
