# Supplementary material for: A Multiple-Choice Task with Changes of Mind
Source: PLoS One. 2012 Aug 16;7(8):e43131. doi: 10.1371/journal.pone.0043131 (PMC3420910; doi:10.1371/journal.pone.0043131)
Supplement: Figure S3 — Attractor model with adapted thresholds compared to frequency distributions of changes for the three participants with most and fewest changes. (PDF) [file pone.0043131.s003.pdf]

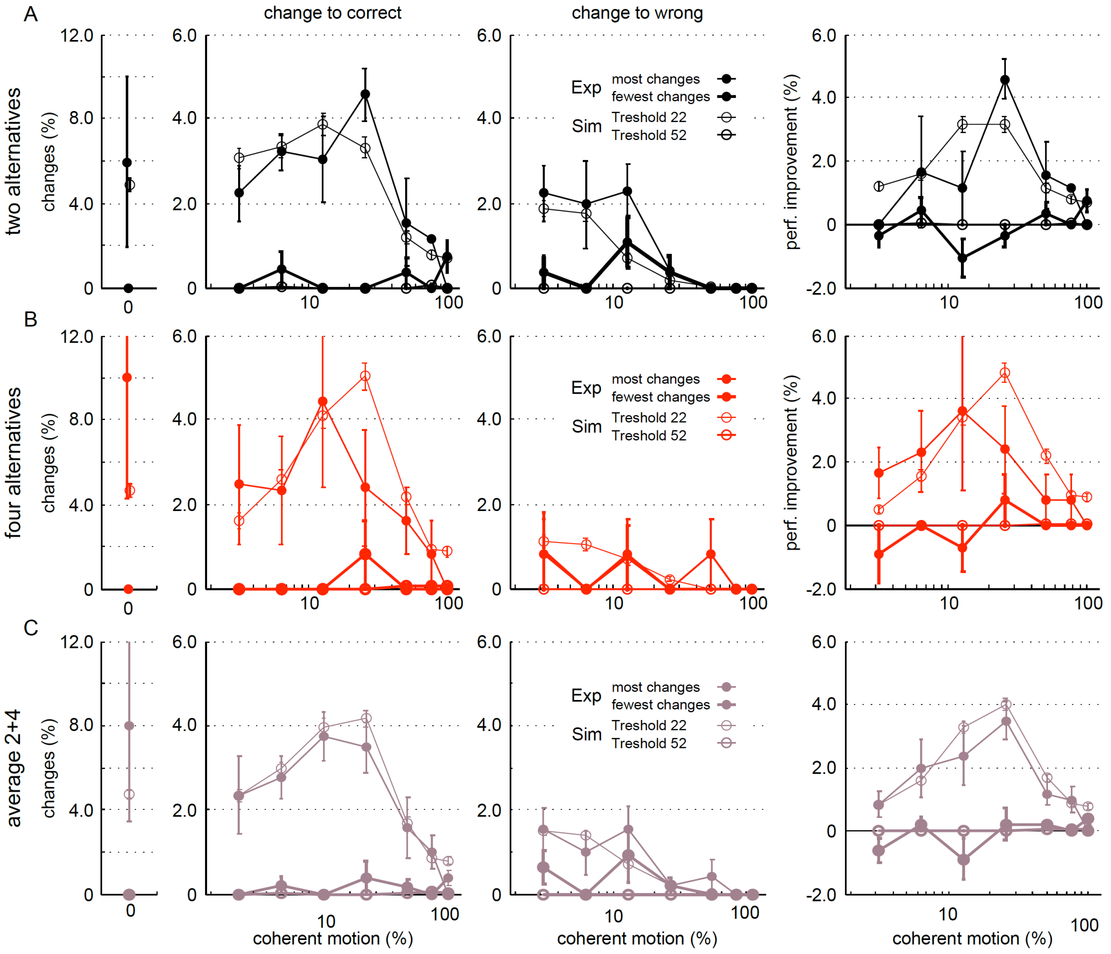


Figure S3: **Attractor model with adapted thresholds compared to coherence dependence of changes for the three participants with most and fewest changes.** Frequency of changes of mind for 0% motion coherence (first column), changes to correct (second column), and changes to wrong (third column) as percentage of all valid trials, as well as the performance improvement (fourth, right column) are shown for two (A) and four (B) choice alternatives, and for the average across the two conditions (C). Filled circles denote the experimental data, open circles simulated data. To model the behavioral data including the percentage of changes for the three participants who changed the most, the decision and change threshold was set to 22 Hz in the attractor model (see main text and Fig. 8). To simulate the behavior of the three participants who showed the fewest number of overall changes, the threshold was set to 52 Hz. All other network parameters were kept constant. Error bars denote SEM.
