# Supplementary material for: A Multiple-Choice Task with Changes of Mind
Source: PLoS One. 2012 Aug 16;7(8):e43131. doi: 10.1371/journal.pone.0043131 (PMC3420910; doi:10.1371/journal.pone.0043131)
Supplement: Figure S4 — Diffusion model fit to behavioral data of the 2-choice condition and 4-choice approximations. (PDF) [file pone.0043131.s004.pdf]

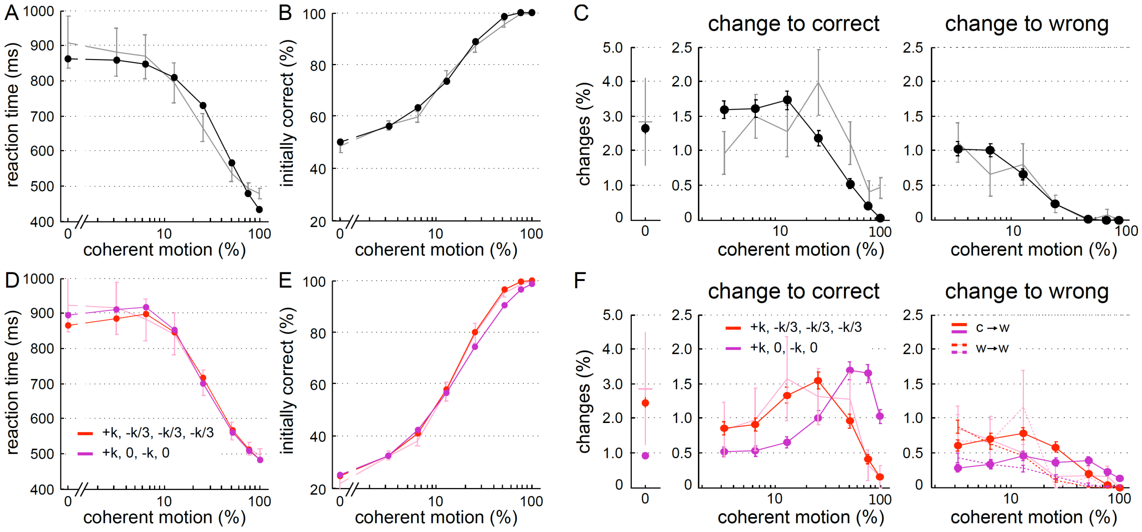

Figure S4: **Diffusion model fit to behavioral data of the 2-choice condition and 4-choice approximations.** (A-C) Resulaj et al. (2009) showed that a diffusion model with a second threshold ΔB and a timeout for changes of mind t_{out} could explain their experimental finding on changes of mind in a binary RDM task. This binary diffusion model is equivalent to a race model in which the two accumulators receive anticorrelated inputs and noise. To test whether such a diffusion model could also account for our experimental results in the 2-choice condition, we performed the same fitting procedure as for the race model (see Methods), only this time with a positive accumulation rate $+k \cdot coh$ for the first accumulator and a negative rate $-k \cdot coh$ for the second accumulator, and perfectly anticorrelated noise to both accumulators. We again repeated the fitting procedure ten times and used the mean estimated parameters to simulate RTs (A) and initial performance (B) of the diffusion model (k = 368±20 Hz/s, Z = -52±1 Hz, and t_{ND} = 287±19 ms (errors denote SD), given normally distributed random increments with mean +/-k and standard deviation of σ = 70 Hz·s$^{1/2}$). The experimental results of Fig. 3 and 4 are plotted in gray for comparison. (C) As for the race model of Fig. 9F, a second independent threshold ΔB = -12±5 Hz and a timeout t_{out} = 371±41 ms for changing determined changes of mind in the diffusion model (errors denote SD). Anticorrelated inputs and noise in the diffusion model correspond to mutual inhibition between accumulators, which decreases the number of changes of mind enormously compared to the race model. This is because the two accumulators will never both approximate the changes of mind threshold simultaneously. Thus,

contrary to the race model, here the value for ΔB is negative, meaning that the threshold for changes of mind is actually lower than the decision threshold for the first decision, and t_{out} is larger than t_{ND}. Taking ΔB and t_{out} as free parameters, estimated through the experimental changes of mind data of the 2-choice condition, the diffusion model fits the experimental data well. Note, however, that a model with an independent second threshold for changes of mind still cannot implicitly account for the changes-speed-accuracy relation found in the experimental data across subjects (see Fig. 5 and 8).

(D-F) While the diffusion model could account for our experimental data in the 2-choice conditions, it is not readily extendable to more choice alternatives. As a first approximation to mutual inhibition between four accumulators, we further tested two adapted versions of the 4-choice race-model assuming positive and negative accumulation rates, but independent noise for all accumulators. ΔB and t_{out} are again free parameters. In particular, we first tried a model similar to the diffusion-race-model hybrid suggested in Churchland et al. (2008), with +k for accumulator #1, -k for accumulator #3, k = 0 Hz/s for accumulators #2 and #4. The simulated behavioral results are displayed in pink in (D-F), with parameters k = 636±50 Hz/s, Z = -59±2 Hz, and t_{ND} = 386±20 ms, ΔB = 20±4 Hz, and a timeout t_{out} = 70±9 ms (errors denote SD). In the second version, we assumed +k for accumulator #1 and –k/3 for the other three accumulators. Results are displayed in red in (D-F), with parameters k = 609±44 Hz/s, Z = -58±1 Hz, and t_{ND} = 386±18 ms, ΔB = 18±4 Hz, and a timeout t_{out} = 81±10 ms (errors denote SD). For comparison, the experimental data of the 4-choice condition (Fig. 3 and 4) is shown in light red. The first model (+k, 0, -k, 0) fails to account for the changes of mind data in the same way as the original race model (Fig. 9E). The second race model extension (+k, -k/3, -k/3, -k/3), however, matches our experimental results quite well. Yet, qualitative differences can still be observed in the RTs (D) for low coherences and the curve shape for erroneous changes of mind (F). In order to produce the experimentally observed order of magnitude for changes of mind, in both models ΔB has to be substantially higher than the first decision threshold and t_{out} very short compared to t_{ND}. This is because the noise in both models is independent for each accumulator just as in the original race model. Implementing a truly diffusion-like model with mutual inhibition and thus anticorrelated noise for four choice alternatives, however, requires a connectionist implementation (Niwa and Ditterich, 2009), which goes beyond the scope of simple conceptual

models. Such a linear connectionist model with explicit mutual inhibition could in principle be able to fit our experimental data of the 4-choice condition even better than the (+k, -k/3, -k/3, -k/3) model with more realistic parameters for ΔB and t_{out}, given the freedom of an independent second threshold. Error bars denote SEM.
