# Supplementary material for: A Multiple-Choice Task with Changes of Mind
Source: PLoS One. 2012 Aug 16;7(8):e43131. doi: 10.1371/journal.pone.0043131 (PMC3420910; doi:10.1371/journal.pone.0043131)
Supplement: Table S2 — Default parameter set used in the integrate-and-fire simulation (attractor model). (PDF) [file pone.0043131.s006.pdf]

Table S 2: Default parameter set used in the integrate-and-fire simulation

Parameter	Value	Parameter	Value
N_{E}	400	N_{I}	100
V_{E}	0 mV	V_{I}	-70 mV
V_{L}	-70 mV	N_{ext}	800
V_{thr}	-50 mV	ν_{ext}	2.4 kHz
V_{reset}	-55 mV	f	0.20
ω_{+}	1.48	ω_{I}	1.125
ω_{-}	0.88	α	0.5 ms^{-1}
C_{m} (excitatory)	0.5 nF	C_{m} (inhibitory)	0.2 nF
g_{m} (excitatory)	25 nS	g_{m} (inhibitory)	20 nS
τ_{ref} (excitatory)	2 ms	τ_{ref} (inhibitory)	1 ms
$g_{\mathrm{AMPA,ext}}$ (excitatory)	2.08 nS	$g_{\mathrm{AMPA,ext}}$ (inhibitory)	1.62 nS
$g_{\mathrm{AMPA,rec}}$ (excitatory)	0.312 nS	$g_{\mathrm{AMPA,rec}}$ (inhibitory)	0.243 nS
g_{GABA} (excitatory)	2.5 nS	g_{GABA} (inhibitory)	1.946 nS
g_{NMDA} (excitatory)	0.6213 nS	g_{NMDA} (inhibitory)	0.4902 nS
$\tau_{\mathrm{NMDA,decay}}$	100 ms	$\tau_{\mathrm{NMDA,rise}}$	2 ms
τ_{AMPA}	2 ms	τ_{GABA}	10 ms
